# Approaches to interim analysis of cancer randomised clinical trials with time to event endpoints: A survey from the Italian National Monitoring Centre for Clinical Trials

**DOI:** 10.1186/1745-6215-9-46

**Published:** 2008-07-25

**Authors:** Irene Floriani, Nicole Rotmensz, Elena Albertazzi, Valter Torri, Marisa De Rosa, Carlo Tomino, Fillipo de Braud

**Affiliations:** 1 Department of Oncology, Istituto di Ricerche Farmacologiche "Mario Negri", Milan, Italy; 2 Division of Epidemiology and Biostatistics, European Institute of Oncology, Milan, Italy; 3 Interuniversity Consortium, Bologna, Italy; 4 Osservatorio Italiano Sperimentazioni Cliniche, AIFA (Italian Drug Agency), Roma, Italy; 5 Unit of Clinical Pharmacology and New Drugs, European Institute of Oncology, Milan, Italy

## Abstract

**Background:**

Although interim analysis approaches in clinical trials are widely known, information on current practice of planned monitoring is still scarce. Reports of studies rarely include details on the strategies for both data monitoring and interim analysis. The aim of this project is to investigate the forms of monitoring used in cancer clinical trials and in particular to gather information on the role of interim analyses in the data monitoring process of a clinical trial. This study focused on the prevalence of different types of interim analyses and data monitoring in cancer clinical trials.

**Methods:**

Source of investigation were the protocols of cancer clinical trials included in the Italian registry of clinical trials from 2000 to 2005. Evaluation was restricted to protocols of randomised studies with a time to event endpoint, such as overall survival (OS) or progression free survival (PFS). A template data extraction form was developed and tested in a pilot phase. Selection of relevant protocols and data extraction were performed independently by two evaluators, with differences in the data assessment resolved by consensus with a third reviewer, referring back to the original protocol. Information was obtained on a) general characteristics of the protocol b) disease localization and patient setting; c) study design d) interim analyses; e) DSMC.

**Results:**

The analysis of the collected protocols reveals that 70.7% of the protocols incorporate statistical interim analysis plans, but only 56% have also a DSMC and be considered adequately planned. The most concerning cases are related to lack of any form of monitoring (20.0% of the protocols), and the planning of interim analysis, without DSMC (14.7%).

**Conclusion:**

The results indicate that there is still insufficient attention paid to the implementation of interim analysis.

## Introduction

### Importance of interim analyses

The simplest approach for evaluating results of a clinical trial is to plan just one statistical analysis at the end of the study, using a fixed-sample size design: planning and conduction are easy, and the methods for estimation are well established. This approach, which is convenient and effective when all observations are available in a short period of time, is less appropriate when data become available sequentially. This is the case in studies on chronic diseases, like cancer, in which recruitment may last many years, so that the first outcomes can be observed when the accrual is still ongoing. In such situations there might be ethical, practical and economic reasons for looking at the data before the planned end of the study.

Data monitoring conducted during a continuing study may focus on performance, data integrity, safety and treatment effect. The assessment of study performance in terms of quality of data, protocol adherence, recruitment rate, is normally performed periodically in an informal way, adopting modalities that can be grouped under the definition of "internal monitoring". In contrast, the tasks of "external monitoring" are to evaluate data integrity, safety and efficacy of treatments and to provide advice on continuing the study as originally planned, or suggesting changes in its conduct, or even on stopping it. This advice is mainly based on trial results, but should take into account the context of information external to the trial available at the moment of the analysis[1].

This process of "interim analysis" is usually conducted by a data and safety monitoring committee (DSMC), usually composed by an independent group of experts in the involved fields (biostatistician, clinical researcher, epidemiologist, clinician with expertise in the disease under investigation) [1,2].

Formal interim analysis offers several advantages, since this approach makes the process of acquiring and disseminating results more efficient and a beneficial treatment can be made available sooner. Ethical reasons play also a role in the decision to stop a trial, since there is a responsibility to minimize the number of subjects treated with an unsafe, ineffective or clearly inferior treatment. On the other hand, conducting an interim analysis may also have drawbacks, since immature results on small numbers of patients will provide imprecise or even biased point and interval estimates of the treatment effect, increasing the error in inferential process [3]: when a clinical trial is closed because a treatment difference has been detected, the estimate of the magnitude of that difference will overstate the "true" value [4]. Finally, trials stopped early are likely to be of small size, and as a consequence their results may lack both statistical precision and credibility, since medical community might remain sceptical, even in case of highly significant results. Therefore, while informal reviews are necessary, the process of repeatedly evaluating data must be done with caution, especially early in the course of a trial when the number of both participants and events related to safety and efficacy are relatively small [5]. For these reasons some investigators strongly recommend that the results of such trials should be treated with scepticism [6].

From the statistical viewpoint, monitoring methods can be classified according to whether the method is frequentist or Bayesian [7] and comprehensive reviews of statistical aspects of monitoring can be found in Whitehead [8], Jennison and Turnbull [9] and Piantadosi [10]. However, regardless of the specific method used, a key issue is that statistical rules are only a part of the question, as they tend to oversimplify the information relevant to the decision that must be taken. The decision to stop a trial before the prespecified final analysis should not be guided only by statistical considerations, but also by practical issues (toxicity, ease of administration, costs, etc.), as well as clinical considerations. For this reason it is preferable to refer to statistical methods as guidelines, rather than rules [11]. Despite these statistical and ethical implications of conducting an interim analysis, information on explicit adoption of planned monitoring is still scarce, basically driven by the published reports of studies, which rarely include details on the strategies for data monitoring and interim analysis. It seemed therefore of interest to investigate the forms of monitoring and interim analysis used in randomised clinical trials in cancer in order to gather information of the quality of research protocols activated in Italy on cancer patients.

## Methods

We assessed protocols available in the OsSC database [12] [see additional file 1] relative to oncological studies submitted to Italian ECs from January 1st 2000 to May 2005 and evaluated and accepted by the coordinating centre by the end of October, 2005. We restricted the evaluation to protocols of randomised studies with a time to event endpoint, such as overall survival (OS) or progression free survival (PFS). A template data extraction form was developed and tested in a pilot phase. Selection of relevant protocols and data extraction were performed independently by two evaluators, with differences in the data assessment resolved by consensus with a third reviewer, referring back to the original protocol.

Information was obtained on a) general characteristics of the protocol (identification number, experimental phase, year of EC opinion release, type of sponsor, involved countries, study objective; b) disease localization and patient setting; c) study design (aim of the study, primary endpoint, number of arms, expected number of events to observe and of patients to randomise, planned number of centres involved in the trial, duration of the study, as well as of the accrual and follow-up periods); d) interim analyses, if present (number, type, objective, timing); e) DSMC, if planned (composition and tasks).

Results were reported using adequate descriptive statistics, such as absolute and relative frequencies for categorical variables and median (interquartile range) for continuous variables, unless otherwise specified.

The association between the use of interim analyses and/or DSMC and potential determinants, such as type of sponsor, involved countries, year of submission, experimental phase and total duration of the study was estimated by a logistic regression model. Results are reported as odds ratios (ORs) and their 95% confidence intervals (95% CIs).

Analyses were performed using SAS (Statistical Analysis System, SAS Institute Inc., Cary, NC, US, Version 8.20) software.

## Results

Seven hundreds ninety-six cancer protocols were identified and manually checked in order to locate the eligible trials.

Figure 1 reports the flow diagram of the selection of relevant protocols [see additional file 2]. From 796 oncological studies found, only 150 (18.8%) were eligible and evaluable for analysis, while the others were excluded for the reasons described in the diagram.

Table 1 describes the characteristics of evaluated trials [see additional file 3].: the majority of the protocols included in this project are international (102, 68.0%), and conducted on solid tumours (129, 86.0%). The more frequently investigated diseases are lung (36, 24.0%) and breast cancers (35, 23.3%). 107 out of 129 (83.0%) of the protocols on solid tumours were conducted in the setting of advanced disease (data not shown). The great majority (138, 92.0%) of the studies were aimed at detecting a difference in efficacy between arms, and the primary endpoint was overall survival in 64 cases (42.7%). The planned median number of patients to randomise and of events to observe was 520 (25°–75° percentiles: 300–820) and 384 (25°–75° percentiles: 218–616), respectively. The expected proportion of events at the end of the study, a good index of patient prognosis, calculated as the ratio of these two latter variables, had a median value of 0.69 (25°–75° percentiles: 0.47–0.78). The median study duration was 42.5 months (25°–75° percentiles: 29–60), given by a median accrual period of 24 months (25°–75° percentiles: 18–36) and a subsequent follow-up of 18 (25°–75° percentiles: 12–30). The median number of experimental centres was 70 (25°–75° percentiles: 35–120), but this information is reported only in 65 protocols.

Table 2 shows the presence of interim analyses and/or of a DSMC [see additional file 4]. 106 (70.7%) protocols planned some form of monitoring, for example on safety, protocol compliance or recruitment rate. When focusing on formal efficacy analysis, this number decreases to 86 (66.2%), because in 20 cases the only checks concerned matters such as safety, feasibility and recruitment rate. The establishment of a DSMC was reported in 98 (65.3%) cases: 84 (56.0%) with a planned interim analysis and 14 (14.7%) in which an interim analysis was not planned. Overall neither form of monitoring took place in 30 out of 150 protocols (20.0%). The median number of interim analyses was 2, ranging from 1 to 9. Among the 86 protocols with an efficacy analysis, 34 (39.5%) planned only 1 interim analysis, 32 (37.2%) 2 interim analysis, 10 (11.6%) 3 interim analysis; and 10 (11.6%) more than 3 analyses,

Table 3 shows the main characteristics of the interim efficacy analyses [see additional file 5]. Of note, among the 86 protocols with an efficacy analysis, in 2 cases the endpoint was not reported, while in 6 (7.0%) it was related to activity, and therefore different from that of the final analysis.

The timing of interim analyses for efficacy was planned according to the proportion of observed events (for example for 2 analyses, the first when 33% of the planned total number of events had been observed and the second at 66%) in 54 (62.8%) protocols, according to the proportion of patients (for example again for 2 analyses after the enrolment of 33% and 66% of the planned total number of patients) in 22 (25.6%) protocols and based on calendar time (for example yearly after the first two years of recruitment) in the remaining 10 (11.6%) cases.

The most frequent type of statistical approach for the analysis was the frequentist method, using the O'Brien and Fleming boundaries: alone in 45 out of 86 (52.3%) studies or together with conditional power in 6 (7.0%). Conditional power alone was used in 6 studies, while a Bayesian approach was used in only one study. In 5 protocols the statistical monitoring method, and therefore the stopping rules, was not specified.

Criteria for stopping were reported in 77 (89.5%) of the protocols, mostly represented by the achievement of a significant difference between arms (52, 60.5%). It is of note that in 2 studies, no mention was made of either the statistical approach or the stopping rules to be adopted.

In 24 (24.5%) out of 98 protocols, the only commitment of DSMC was safety. Efficacy was considered in 68 protocols (69.4%). In one study it was the only task of DSMC, while in the remaining 67 the DSMC was in charge of monitoring both safety and efficacy. The composition and frequency of DSMC meetings are reported only in 34 (34.7%) and 40 (40.8%) protocols, respectively. The DSMC was stated to be independent in 80 (81.6%) of the protocols. The committee usually consisted of 3 or 4 members, always including a statistician, In 8 cases sponsor representatives could participate as non voting-member. The frequency of the meetings was generally twice a year.

Table 4 shows the results of univariate and multivariate logistic models, assessing the association among selected characteristics of the study protocols and the presence of both interim analysis and DSMC [see additional file 6]. In both models, the only variable associated with the presence of an interim analysis was the international organization of the study, accounting for an odds ratio of 3.72 (95% CI 1.70–8.13) and of 4.75 (95% CI 1.38–16.4), respectively. The most important factors associated with the presence of DSMC are a commercial sponsor (OR 4.37, 95% CI 1.38–13.9) and international collaboration (OR 10.9, 95% CI 3.06–38.6).

## Discussion

This project was aimed at assessing the prevalence of interim analyses and DSMC in randomised clinical trials in cancer, using as source the database including all phase II-III trials submitted to Italian ECs from January 2000 to May 2005. The Italian registry of clinical trials gave an unique opportunity to obtain this information and allowed a critical appraisal of the statistical designs utilized in current cancer clinical trials in Italy.

The reason for choosing a protocol registry rather than literature data, stems from the observation that in published papers the quality of details relative to the description of statistical methods is often scarce, and it is possible that, despite the accuracy of such a search strategy, the information regarding the approaches adopted for monitoring clinical trials is not completely or accurately captured. Moreover, even when reported, statistical analysis of cancer published trials usually concerns protocols designed years before the study publication and, as a consequence, data derived from even the most recent published literature may not be completely appropriate to represent the currents trend of interim analysis and DSMC use. Finally, since Italian registry includes several international protocols, it may also be considered an important source of information generalizable also to ongoing researches in European countries.

The most important conclusion arising from this study is that at present around 30% of the protocols do not incorporate any form of interim analysis plan, and only 56% of protocols can be considered adequately planned for monitoring the trial. Although this result suggests an increasing use of monitoring tools with respect to the past, it is still not completely satisfactory. Despite the availability of several statistical methods for interim analysis, the almost uniquely approach is the frequentist method while the Bayesian approach is rarely considered, although in the context of monitoring it would be more useful for its characteristics of flexibility in incorporating external evidence.

Interim analysis plans are still described very infrequently, even in the more recent protocols, denoting insufficient attention to this issue not only by the researchers, but also by ethical committees who have a responsibility to consider the ethical and scientific aspects of the submitted studies. In this context there is a discrepancy between the perceived importance of data monitoring boards and their presence (65.3% of the protocols) and the lack of information regarding their composition and their role. It is encouraging that the timing of interim analysis was usually related to the number of events and almost half of the trials adopted no more than one planned interim analysis, thus reducing the risk of biased estimation.

Interim analyses play a fundamental role in the balance between the need of timely information regarding the treatment effect and the control of false positive errors and estimation bias. Moreover, the adoption of a statistical approach for data monitoring, no matter of which type is chosen, effectively protects the study from the risk of incorrect early stopping. If no stopping rule is adopted, the probability of early stopping with a higher estimation is noticeably increased [5,13].

Stabilization of the estimates happens when a substantial amount of events have occurred. The finding that estimation bias tends to be reduced, as expected, when the observed number of events is closer to the planned size, is particularly important for cancer clinical trials for non-statistical reasons. With time to event endpoints, a potential problem with stopping a trial early is that the early survival experience with short follow-up may not accurately reflect the complete experience with time. A new treatment may be very toxic, leading to a few early deaths, but may also have much better long-term results than the standard treatment. Overall, the new treatment may be viewed as better than the standard, but an early look at the data may suggest stopping for lack of efficacy. The opposite is also possible, since early suggestions of treatment efficacy may decline over time.

Another factor that should be considered is the scientific and ethical relationship that links the decision of stopping a trial early with its implication for ongoing trials, addressing the same clinical question, or when a confirmatory trial is planned. These issues were well quantified by a systematic review of Montori et al.^6^, analysing 143 randomised clinical trials (RCTs) stopped early for benefit, and generally published in high-impact medical journals and were industry-funded drug trials. The proportion of all RCTs published that were stopped early for benefit increased from 0.5% in 1990–1994 to 1.2% in 2000–2004. On average, RCTs recruited only 63% of the planned sample and stopped after a median of 13 months of follow-up, 1 interim analysis, and when a median of only 66 patients had experienced the end point driving study termination (event). The median risk ratio among truncated RCTs was high: 0.53 (25°–75° percentiles: 0.28–0.66). One hundred thirty-five (94%) of the 143 RCTs did not report at least 1 of the following: the planned sample size the interim analysis after which the trial was stopped, whether a stopping rule informed the decision, or an adjusted analysis accounting for interim monitoring and truncation (n = 129).

## Conclusion

Our results add important information relative to the methodological quality of clinical studies. Research conducted so far took into consideration only published report and achieved qualitatively similar findings. The DAMOCLES working party^2 ^addressed several different issues, using different methodological approaches: systematic literature reviews of DSMC, small group processes in decision-making; sample surveys of reports of RCTs, recently completed and still ongoing RCTs and policies of major organisations involved in RCTs; case studies of selected DSMCs; and interviews with experienced DSMC members. The results of these studies indicated that only about a quarter of main RCT reports mention use of a DSMC and wide variation exists in the structure and organisation of DSMCs, with little guidance on how they should operate. Our research suggests that there is an increased proportion of studies reporting use of DSMC, but is qualitatively in agreement with these conclusions. The study clearly indicates that much has still to be done in making trialists aware of the statistical analyses that should be implemented, of the impact of results of interim analyses on the final decision to be taken, and of the role of DSMC. Our findings may be also be of help in the identification of the questions to be addressed by further research for improving organisation and conduction of clinical trials. In this sense, the survey of Italian protocols seems of particular interest. Although we are aware that the results may be valid for the Italian research context and not totally generalisable to other countries, we think that this enquiry represents a good basis for debating the issues on how to improve monitoring of clinical trials. It also emphasises the importance of the adoption of national registries and encourages the replication of this kind of research in other countries were national registries of clinical trials are available.

## Supplementary Material

Additional file 1 Box: The "Osservatorio Nazionale sulla Sperimentazione Clinica dei Farmaci". The Box reports details on Osservatorio Click here for file

Additional file 2 Figure 1 – Search flow diagram. The figure shows the reasons for protocols selection Click here for file

Additional file 3 Table 1 – Description of 150 evaluable trials. The table gives information on selected characteristics of evaluable trials Click here for file

Additional file 4 Table 2 – Presence of interim analyses and of a DSMC (n = 150). The table provides details on presence of interim analysis and DSMC Click here for file

Additional file 5 Table 3 – Characteristics of the interim efficacy analyses (n = 86). The table provides details on characteristics of interim analysis Click here for file

Additional file 6 Table 4 – Relationship among presence of interim analysis/DSMC and selected protocol. characteristics – Odds Ratios (95% Wald Confidence Intervals) at multivariate analysis. The table provides details on logistic analysis for evaluating the association of selected protocol characteristics and presence of interim analysis plan and DSMC. Click here for file
