# Supplementary material for: Approaches to interim analysis of cancer randomised clinical trials with time to event endpoints: A survey from the Italian National Monitoring Centre for Clinical Trials
Source: Trials. 2008 Jul 25;9:46. doi: 10.1186/1745-6215-9-46 (PMC2533282; doi:10.1186/1745-6215-9-46)
Supplement: Additional file 1 — Box: The "Osservatorio Nazionale sulla Sperimentazione Clinica dei Farmaci". The Box reports details on Osservatorio [file 1745-6215-9-46-S1.pdf]

The “Osservatorio Nazionale sulla Sperimentazione Clinica dei Farmaci”

Since 2000, the Italian Ministry of Health has established that all data concerning clinical trials with medicines have to be collected in a centralized electronic registry, the “Osservatorio Nazionale sulla Sperimentazione Clinica dei Farmaci - OsSC” (National Monitoring Centre on Clinical Research with Medicines), for approval by the Italian Ethics Committees (ECs) and the Competent Authorities (Istituto Superiore di Sanità – the leading scientific and technical public body of the Italian National Health Service - and AIFA – the Italian Agency of Medicines). The OsSC has thus become a unique instrument to improve coordination and surveillance of current pharmacological research in Italy[12].

The informative support of the OsSC consists of three on-line registers (for the local ECs, a Register of private clinical sites and a register of clinical trials) that form one single database of clinical trials planned to be updated and consulted electronically. The Ministry of Health, the local ECs, the Sponsors, the Regions and the autonomous Provinces can access the information on these registers, according to their organizational needs. Data entry activity has been mainly entrusted to Sponsors, CROs (Contract Research Organisations, when working on behalf of sponsors), Ethics Committees responsible for the assessment of clinical trial applications and documents as well as to Istituto Superiore di Sanità and AIFA.

Since December 2005 the OsSC has also been open for general public consultation.

The OsSC may offer an excellent opportunity for conducting research on clinical trial strategies, by investigating the type and relevance of current clinical research questions, as well as the quality of the design and other methodological criteria adopted by researches currently ongoing in Italy. The project reported here is one of the first to take advantage of the registry.
