# Supplementary figures and images for: Approaches to interim analysis of cancer randomised clinical trials with time to event endpoints: A survey from the Italian National Monitoring Centre for Clinical Trials
Source: Trials. 2008 Jul 25;9:46. doi: 10.1186/1745-6215-9-46 (PMC2533282; doi:10.1186/1745-6215-9-46)

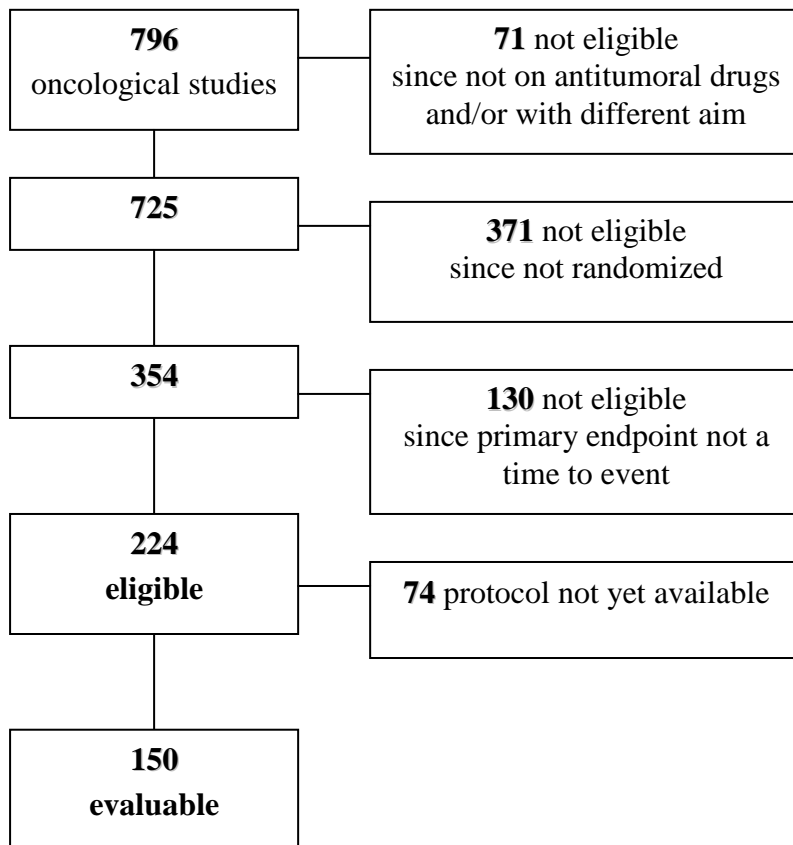

Supplement: Additional file 2 — Figure 1 – Search flow diagram. The figure shows the reasons for protocols selection [file 1745-6215-9-46-S2.pdf]
