# Supplementary material for: Approaches to interim analysis of cancer randomised clinical trials with time to event endpoints: A survey from the Italian National Monitoring Centre for Clinical Trials
Source: Trials. 2008 Jul 25;9:46. doi: 10.1186/1745-6215-9-46 (PMC2533282; doi:10.1186/1745-6215-9-46)
Supplement: Additional file 3 — Table 1 – Description of 150 evaluable trials. The table gives information on selected characteristics of evaluable trials [file 1745-6215-9-46-S3.pdf]

	N (%)
**Year**	
2000	17 (11.3)
2001	21 (14.0)
2002	18 (12.0)
2003	38 (25.3)
2004	39 (26.0)
2005	17 (11.3)
**Involved countries**	
Only Italy	37 (24.7)
Europe	11 (7.3)
Worldwide	102 (68.0)
Not specified	-
**Phase**	
II	12 (8.0)
III	138 (92.0)
**Sponsor**	
Non profit	47 (31.3)
Profit	103 (68.7)
