# Supplementary material for: Approaches to interim analysis of cancer randomised clinical trials with time to event endpoints: A survey from the Italian National Monitoring Centre for Clinical Trials
Source: Trials. 2008 Jul 25;9:46. doi: 10.1186/1745-6215-9-46 (PMC2533282; doi:10.1186/1745-6215-9-46)
Supplement: Additional file 4 — Table 2 – Presence of interim analyses and of a DSMC (n = 150). The table provides details on presence of interim analysis and DSMC [file 1745-6215-9-46-S4.pdf]

		N	%
**Presence of interim analysis**		**106**	**70.7**
≥1 formal efficacy analysis		86	66.2
Only safety analysis		20	33.8
**Presence of DSMC**		**98**	**65.3**
**Interim analysis**	**DSMC**		
Yes	Yes	84	56.0
	No	22	14.7
No	Yes	14	9.3
	No	30	20.0
