# Supplementary material for: Approaches to interim analysis of cancer randomised clinical trials with time to event endpoints: A survey from the Italian National Monitoring Centre for Clinical Trials
Source: Trials. 2008 Jul 25;9:46. doi: 10.1186/1745-6215-9-46 (PMC2533282; doi:10.1186/1745-6215-9-46)
Supplement: Additional file 5 — Table 3 – Characteristics of the interim efficacy analyses (n = 86). The table provides details on characteristics of interim analysis [file 1745-6215-9-46-S5.pdf]

	N	%
**OBJECTIVE**		
**Same to primary analysis**	**74**	**86.0**
**Different from primary analysis**	**12**	**14.0**
- Activity	6	7.0
- Time to event different to primary endpoint	4	4.6
- Not reported	2	2.3
**STATISTICAL APPROACH**		
**Specified**	**81**	**94.2**
- Frequentist, OBF*	45	52.3
- Frequentist, OBF+CP**	6	7.0
- Frequentist, CP	6	7.0
- Frequentist, Haybittle-Peto	3	3.5
- Frequentist, TT#	1	1.2
- Frequentist, other	19	22.1
- Bayesian	1	1.2
**Not specified**	**5**	**5.8**
**REASON FOR STOPPING**		
**Reported**	**78**	**89.5**
- Superiority/inferiority of experimental arm	52	60.5
- Futility	7	8.1
- Superiority of experimental arm	6	7.0
- Superiority/futility of experimental arm	5	5.8
- Inferiority of experimental arm	3	3.5
- Superiority/inferiority of experimental arm and safety	2	2.3
- Futility (stop enrollment)	2	2.3
**Not scheduled**	**1**	**1.2**
**Not reported**	**8**	**9.3**
**TIMING**		
Event	54	62.8
Patient	22	25.6
Calendar time	10	11.6

* Alpha-spending function with O’Brien & Fleming boundaries

** Conditional power #Triangular test
