# Supplementary material for: Approaches to interim analysis of cancer randomised clinical trials with time to event endpoints: A survey from the Italian National Monitoring Centre for Clinical Trials
Source: Trials. 2008 Jul 25;9:46. doi: 10.1186/1745-6215-9-46 (PMC2533282; doi:10.1186/1745-6215-9-46)
Supplement: Additional file 6 — Table 4 – Relationship among presence of interim analysis/DSMC and selected protocol. characteristics – Odds Ratios (95% Wald Confidence Intervals) at multivariate analysis. The table provides details on logistic analysis for evaluating the association of selected protocol characteristics and presence of interim analysis plan and DSMC. [file 1745-6215-9-46-S6.pdf]

	Univariate analysis OR (95% CI)	*Multivariate analysis* OR (95% CI)
**Interim analysis**		
Profit sponsor	1.38 (0.66-2.91)	1.42 (0.44-4.57)
International collaboration	3.72 (1.70-8.13)	4.75 (1.38-16.4)
Year of submission	1.13 (0.90-1.42)	0.91 (0.67-1.24)
Experimental phase III	1.81 (0.54-6.06)	0.87 (0.18-4.31)
Study duration (for each 1 year interval)	1.22 (0.98-1.52)	1.50 (1.10-2.07)
Number of patient (for each hundred)	1.04 (0.99-1.09)	1.00 (0.95-1.04)
**DSMC**		
Profit sponsor	3.20 (1.55-6.58)	4.37 (1.38-13.9)
International collaboration	11.5 (4.80-27.7)	10.9 (3.06-38.6)
Year of submission	1.30 (1.04-1.62)	1.09 (0.78-1.53)
Experimental phase III	2.89 (0.87-9.62)	2.86 (0.58-14.0)
Study duration (for each 1 year interval)	0.91 (0.77-1.08)	1.10 (0.84-1.45)
Number of patient (for each hundred)	1.10 (1.02-1.19)	1.06 (1.00-1.13)
